# TERT—Regulation and Roles in Cancer Formation

**DOI:** 10.3389/fimmu.2020.589929

**Published:** 2020-11-19

**Authors:** Marta Dratwa, Barbara Wysoczańska, Piotr Łacina, Tomasz Kubik, Katarzyna Bogunia-Kubik

**Affiliations:** ^1^ Laboratory of Clinical Immunogenetics and Pharmacogenetics, Hirszfeld Institute of Immunology and Experimental Therapy, Polish Academy of Sciences, Wroclaw, Poland; ^2^ Department of Computer Engineering, Faculty of Electronics, Wrocław University of Science and Technology, Wroclaw, Poland

**Keywords:** telomerase reverse transcriptase, cancer progression, *TERTp* mutations, telomere maintenance mechanisms, *TERT* structural variants, *TERT* epigenetic alterations, *TERT* transcriptional activators and repressors

## Abstract

Telomerase reverse transcriptase (TERT) is a catalytic subunit of telomerase. Telomerase complex plays a key role in cancer formation by telomere dependent or independent mechanisms. Telomere maintenance mechanisms include complex *TERT* changes such as gene amplifications, *TERT* structural variants, *TERT* promoter germline and somatic mutations, *TERT* epigenetic changes, and alternative lengthening of telomere. All of them are cancer specific at tissue histotype and at single cell level*. TERT* expression is regulated in tumors *via* multiple genetic and epigenetic alterations which affect telomerase activity. Telomerase activity *via TERT* expression has an impact on telomere length and can be a useful marker in diagnosis and prognosis of various cancers and a new therapy approach. In this review we want to highlight the main roles of *TERT* in different mechanisms of cancer development and regulation.

## Introduction

**Figure 1:**
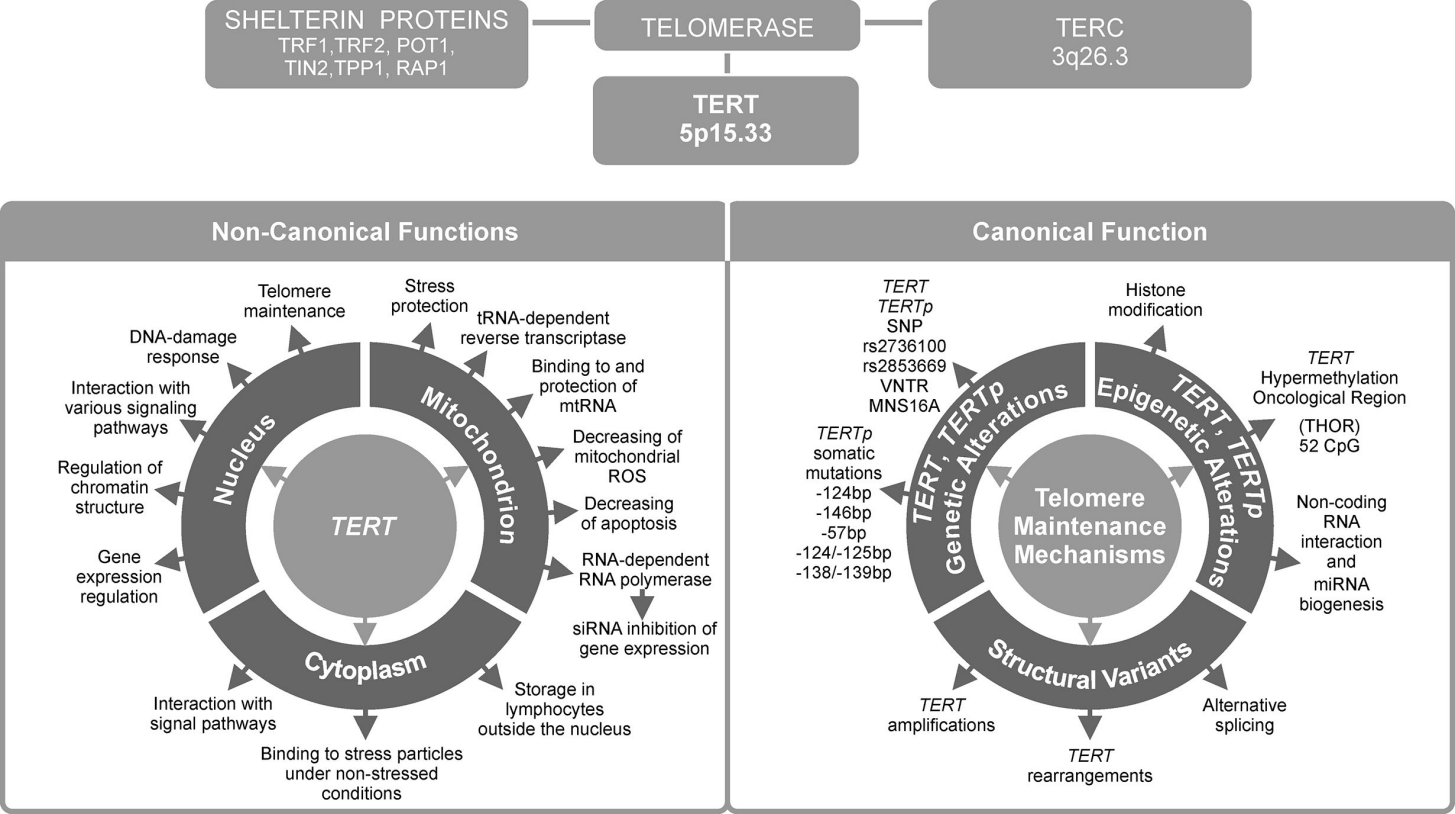
Telomerase reverse transcriptase (TERT) is the most important telomerase subunit and plays a major role in telomerase activity and in other telomere-unrelated processes in cancer development. Telomerase is a complex reverse transcriptase that comprises, besides TERT, an RNA template for telomere repeats (TERC), and a group of proteins called shelterin complex (upper panel). While the primary function of TERT is telomere lengthening (canonical function, lower panel, on the right), there are also other, telomere-unrelated functions (non-canonical functions, lower panel, on the left).

In most human cancers, telomerase is reactivated during carcinogenesis by expression of the catalytic subunit telomerase reverse transcriptase (TERT). TERT plays a key role in cancer formation, ensuring chromosomal stability by maintaining telomere length, and allowing cells to avert senescence. It constitutes a limiting factor for formation of the telomerase complex in cancer cells (1). TERT is one of two major components of the larger telomerase complex, which extends telomeres by adding specific short repetitive DNA sequences. These tandem repeats are bound by the shelterin complex, which is composed of six proteins: telomere repeat factor 1 and 2 (TRF1, TRF2), protection of telomeres 1 (POT1), TRF1-interacting nuclear protein 2 (TIN2), tripeptidyl peptidase I (TPP1), and repressor/activator protein 1 (RAP1) (**Figure 1**) (2). The Shelterin complex plays a fundamental role in protecting chromosome ends and in telomere length regulation (3, 4).

The *TERT* gene is situated at chromosome 5p15.33 in humans, and is an integral and essential part of the telomerase holoenzyme. *TERT* gene is 42 kb long and consists of 15 introns and 16 exons with a 260 bp promoter core (5). The reverse transcriptase domain is encoded by 5–9 exons. The TERT transcript can be spliced into 22 isoforms (6). *TERT* promoter (*TERTp*) region contains GC boxes that bind the zinc finger transcription factor Sp1, which increases *TERT* transcription, and E-boxes that bind both transcriptional enhancers and repressors. *TERTp* lacks a TATA box but it contains binding sites for many different transcription factors (7).

Another major component of the telomerase complex is telomerase RNA component (TERC). It is an RNA sequence, which functions as a template for synthesis of telomeres by TERT. These two main components of telomerase are accompanied by a host of auxiliary proteins, including dyskerin (DKC1), telomerase Cajal body protein 1 (TCAB1), non-histone chromosome protein 2 (NHP2), nucleolar protein 10 (NOP10), glycine arginine rich 1 (GAR1), heat shock protein 90 (HSP90) and serine and arginine rich splicing factor 11 (SRSF11) (8). This complex is essential for maintaining telomere homeostasis, which is crucial in regulation of aging and cancer development (9).

Over 80% of tumors adopt various regulatory strategies, known as telomere maintenance mechanisms (TMMs). They maintain telomere length by reactivating telomerase, and therefore are known as TERT canonical functions (10). Individual TMMs are specific for cancer type, tissue histotype, and cell lines. The most important TMMs are (1) *TERT* gene rearrangements and *TERT* and *TERC* gene amplification, (2) *TERTp* somatic mutations, (3) epigenetic alterations, (4) transcription factor binding, (5) polymorphic variants within *TERT* gene body and *TERTp*, and (6) alternative splicing (**Figure 1**). Each of these mechanisms will be described in detail in subsequent sections of this manuscript.

Approximately 10–15% of tumor cells acquire immortality through a telomerase-independent mechanism, namely alternative lengthening of telomeres (ALT) (11). On the other hand, the so called non-defined telomere maintenance mechanism (NDTMM) are activated when both telomerase (or TERT) expression and ALT are absent (10, 12). While telomere lengthening is considered a major function of telomerase, it can also modulate expression of various genes, such as nuclear factor κ-light-chain-enhancer of activated B cells (NF-κB) and Wnt/β-catenin signaling pathway genes (13, 14). Such alternative, non-telomere-related roles are known as non-canonical functions of TERT. They will be presented in the last two chapters of this review (**Figure 1**), together with potential consequences of TERT telomere-unrelated functions for the development of anti-cancer strategies and applications of TERT as a potential therapeutic target.

## Chromosomal Rearrangements

Chromosomal rearrangements are a type of mutation that results in a change in chromosome structure. They may involve duplications, amplifications, insertions, interchromosomal changes, inverted orientations, or deletions (15). A concept associated with chromosomal rearrangements is copy number variation (CNV). CNV describes the fact that some sections of the genome may be repeated and the number of these repeats may be different between individuals. CNVs involve 50 bp to over 1,000,000 bp fragments of gene regulatory regions (16). They are associated with gene expression and phenotype by affecting gene copy number (17). Chromosomal rearrangements may affect *TERT* gene copy number and are a known TMM. They may involve insertion of active enhancers close to the *TERT* gene and increasing *TERT* expression. A common process is *TERT* amplification, which can arise from telomere dysfunction (18). It results from a dysfunctional telomere, promoting fusion of chromosome ends, and subsequently forming a dicentric chromosome (19). Several studies showed that chromosomal rearrangements at the *TERT* locus may be associated with cancer development and as was observed, e.g., in the case of neuroblastoma (20–22). Furthermore, a major study specifically focusing on *TERT* gene amplification found it to occur in many cancers, such as esophageal, ovarian cancer, and squamous cell carcinoma (12). In addition, other authors found telomerase activity to be the highest in tumors with *TERT* amplification (22, 23). Gay-Bellile et al. observed increased number of *TERT* gene copies in breast cancer cells, and upregulation of *TERT* gene was associated with worse prognosis in breast cancer, thyroid carcinoma, and lung adenocarcinoma (24). This suggests that *TERT* rearrangement could be a critical step in cancer development.

## *TERT* Promoter Hot-Spot Mutations

*TERT* somatic mutations are the most common non-coding mutations in human cancer cells. While they are documented to occur in the coding region, they are far more common in the promoter region. Some *TERTp* mutations were shown to affect *TERT* expression, telomere length and telomerase activity by abrogating telomerase silencing (25). *TERTp* mutations occur in specific clinical and phenotypic subtypes of various cancers and cell lines, and recurrent mutations have been identified in 19% of cancers (26). In cancer cells, *TERTp* mutations are generally associated with higher *TERT* expression level.

The two most common *TERTp* mutations are C>T transitions, located at -124 bp, and -146 bp from the transcription start site (TSS). They are also referred to as C228T and C250T, respectively (27, 28). These mutations result in an 11 bp nucleotide fragment providing a new consensus binding site for E-twenty-six (ETS) transcription factors (29). Many other somatic mutations were detected that occur in the *TERTp* in cancer, although less frequently than C228T and C250T and they also may contribute to increased *TERT* transcription. A group of CC>TT substitutions, located at −124/−125 and −138/−139 bp relative to the TSS, result in an ETS binding site in skin cancers (30). In melanoma patients, the −138/−139 mutation correlated with more adverse survival (31). In basal cell carcinoma, Maturo et al. observed additional *TERTp* alterations other than the recurrent *TERTp* hotspot mutations (32).

*TERTp* mutations were found in several tumor types with different frequencies. Generally, two types of tumors can be distinguished: those with low and high proliferative potential (33). Tumors with high levels of *TERTp* mutation, such as, melanoma, glioblastoma, bladder cancer or hepatocellular carcinoma (somatic mutation levels of 64–80%, ~84%, ~65%, and 32–45%, respectively) are characterized by low proliferative potential (28, 33–36). Tumors with low or undetectable level of *TERTp* mutation have high proliferative potential, e.g., breast cancer 0.9% (37), testicular cancer 3% (38), intestinal cancer (34) and acute myeloid leukemia and non-Hodgkin’s lymphoma (39, 40). It is important to note that *TERTp* mutation was not detected in hematological cell lines cultured *in vitro* (41), as well as in a group of patients with hematological malignances, with the exception of mantle cell lymphoma patients (42). In the case of cancers with low proliferative potential, *TERTp* mutation is considered a late tumorigenic event (33). In some other cancers, e.g., basal cell carcinoma, *TERTp* mutations may appear as a result of environmental factors, such as contact with carcinogens, in which case it is considered as an early tumorigenic event (10, 26). *TERTp* mutations are thought to contribute to tumorigenesis in two distinct phases. In the first phase, *TERTp* mutations heal the shortest telomeres, thus extending life span of cells containing them, but they fail to avert general telomere shortening. This leads to the second phase, where the critically short telomeres result in genomic instability, causing further increase in telomerase expression needed for continued cell proliferation (43).

Another interesting aspect of *TERTp* mutation is the possible cooperation with mutations, such as those in genes coding for BRAF, FGFR3, and IDH (44–48). BRAF is a serine/threonine kinase and its mutation results in activation of the mitogen-activated protein kinase (MAPK) and/or phosphatidylinositol 3-kinase–serine threonine protein kinase (PI3K-AKT) pathways. This leads to upregulation of the ETS system and induction of *TERT* expression. Out of a variety of *BRAF* mutations, *V600E* (a glutamic acid to valine substitution) is the most frequent. This mutation leads to increased GABPA-GABPB complex formation and activation of *TERT* expression (29, 49). Co-existence of *TERTp* mutation and *V600E* is associated with poor prognosis in patients with thyroid cancer, particularly papillary thyroid cancer (8, 50). Fibroblast growth factor receptor 3 (*FGFR3*) is another example of genetic alterations interacting with *TERTp*. Its mutation is well described in urothelial carcinoma (51). FGFR3 belongs to the tyrosine kinase receptor family and stimulates the RAS-mitogen-activated protein kinase and PI3K-AKT pathways. *TERTp* and *FGFR3* mutations are more often present together than alone (47). Co-occurrence of these mutations may support creation of tumors with poor prognosis (10). Additionally, tumors with *TERTp* and/or *FGFR3* mutations had shorter telomeres when compared to tumors without these mutations (47). Malignant gliomas, acute myeloid leukemia and cholangiocarcinoma, are often associated with mutations in isocitrate dehydrogenase 1 and/or 2 (*IDH1* and/or *IDH2*) (52). These somatic mutations occur at arginine residues of the IDH active site (namely, IDH1^R132H^, IDH2^R140Q^, and IDH2^R172K^) (53). According to Diplas et al., *TERTp* and *IDH* mutation status can be used together to classify over 80% of all diffuse gliomas (54). A previous study suggested that presence of *TERTp* mutation and additional 1p/19q co-deletion and also mutation within the *IDH* gene led to a better response to chemotherapy and better outcome in glioma patients (55).

In conclusion, *TERTp* mutation status, alone or in combination with mutations in other genes, can be used to characterize distinguish various types of tumors, as well as predict prognosis and outcome. While *TERTp* mutation status appears to significantly impact cancer development, some cancers, such as prostate, lung, breast, colorectal, and hematological malignancies display telomerase activity, even though they contain few *TERTp* mutations (24, 39, 40, 56, 57). Consequently, other undefined or epigenetic mechanisms of *TERT*-upregulating are expected to exist.

## Epigenetic Modifications

### DNA Methylation

Epigenetics describes stable, and possibly heritable changes in activity and expression, which are not associated with any underlying changes in DNA sequence (58). DNA methylation is a common epigenetic mechanism that is essential for regulation of gene expression. It occurs primarily at non-coding regions of DNA characterized by high frequency of CG repeats. Such regions, called CpG islands, are most commonly found in gene promoters. 60–70% of genes contain promoters with these CpG islands (59).

Tissue-specific DNA hypo- or hypermethylation is considered to be important in regulation of gene expression during development. Such tissue-specific DNA hypermethylation is present at promoters rich in CpG islands (60, 61). Promoter DNA methylation is ubiquitous in human cells and is one of the most commonly encountered mechanisms of gene expression regulation. Promoter methylation generally causes gene silencing by interfering with transcription factor bindings sites. Therefore, promoters of actively transcribed genes are normally unmethylated (62). However, DNA hypermethylation may occur at introns/exons (rather than promoters) of actively transcribed genes, as well as at intra- and intergenic enhancers (63). Having an important role in tissue-specific regulation of transcription, DNA hypermethylation may be considered as a marker for a broad variety of diseases and cancers (64, 65).

**Figure 2:**
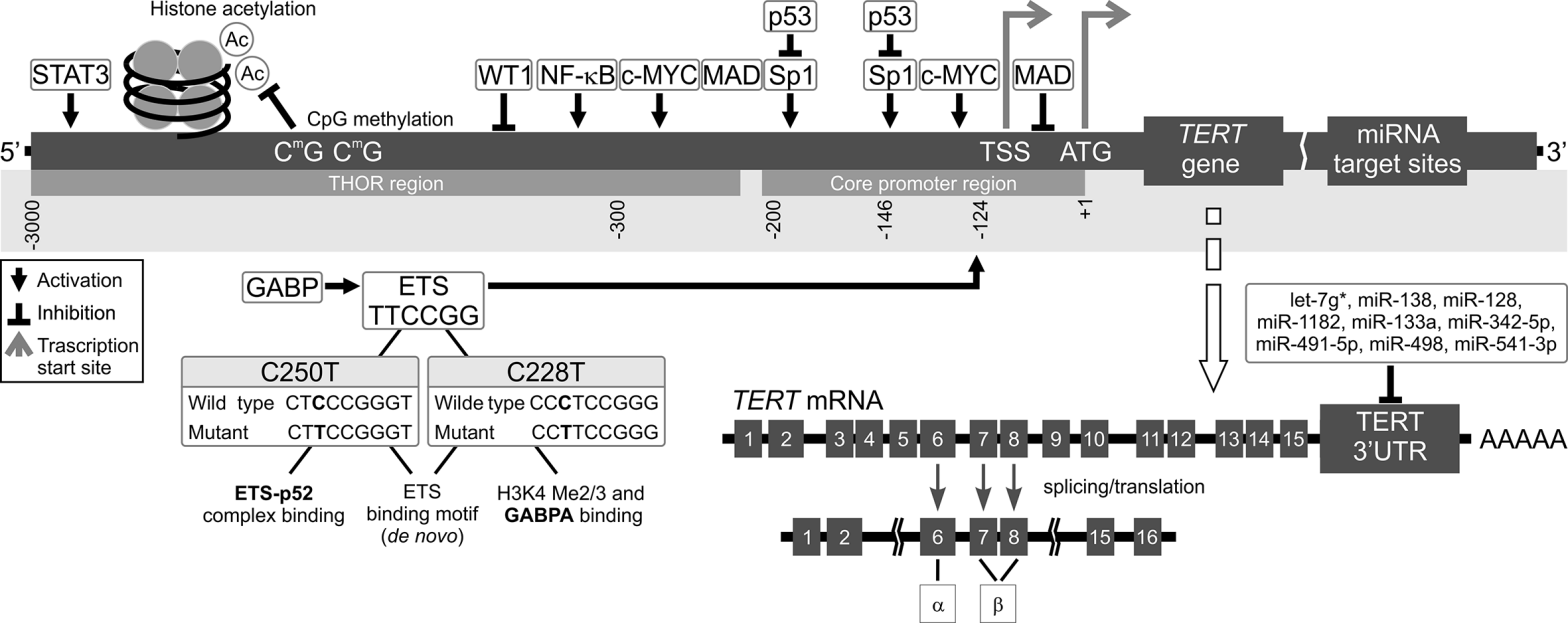
Mechanisms of *TERT* transcription regulation. The figure shows various mechanisms regulating *TERT* expression at the transcriptional level. Transcription factors: activators (e.g., c-MYC, SP1, STAT3, NF-κB, and ETS), repressors (e.g., MAD, p53, and WT1), and their respective binding sites are shown. Binding of these transcriptional agents to *TERT* could be controlled by DNA methylation (CpG sites) in the TERT Hypermethylation Oncological Region (THOR). Two main hotspot mutations within *TERTp*, -146C > T (C250T) and -124C > T (C228T) upstream of the transcription start site (TSS) generate new E-twenty-six (ETS) binding sites, leading to GABP recruitment and, eventually, *TERT* transcription. Alternatively spliced variants of *TERT*, which do not have telomerase activity, could be also generated. Most tissues and organs express no or very low levels of *TERT* mRNA, dependent on histone markers that are correlated with passive or active transcription in many cells. The figure also shows different miRNAs at the 3’UTR that inhibit translation of *TERT*.

Promoter methylation is also a major regulatory element of *TERT* expression, correlating both with *TERT* mRNA levels and telomerase activity (66). An approximately 300 bp part of *TERTp* situated on either site of the TSS is unmethylated in actively transcribed *TERT*. However, Castelo-Branco et al. and, more recently, Lee et al. documented that hypermethylation of the *TERT* gene correlates with telomerase activity in different types of cancers (67–69). A study on patients with pediatric brain tumors brought to light a new group of 5 CpG islands located upstream of the TSS, which were hypermethylated and correlated with *TERT* expression. On the other hand, healthy tissues without *TERT* expression did not have this hypermethylation (59). This pattern is counter to the generally established functions of DNA methylation (63). Lee et al. discovered that it is due to presence of a new, larger region known as the *TERT* Hypermethylated Oncological Region (THOR). It is located distal to the TSS and is composed of 52 CpG islands (69, 70). This means that there are two regions of *TERTp* regarding methylation status in telomerase-positive cells: the unmethylated proximal *TERT* core promoter, which is where transcription factors are usually bound, and the hypermethylated THOR, located further away from the core promoter (67, 69, 71) (**Figure 2**). The unusual nature of THOR methylation is due to it acting as a transcription repressor in its unmethylated state. Recently, several authors documented an association between THOR hypermethylation and cancer progression coupled with *TERT* upregulation in pancreatic and gastric cancers (72, 73). Interestingly, both THOR and the *TERTp* region proximal to TSS were mostly unmethylated in normal thyroid tissue (49).

Regarding *TERTp* mutation status, it appears that it does interfere with effects of THOR hypermethylation in cancers where *TERTp* mutation is common. Furthermore, presence of both of these factors may have a synergistic effect on *TERT* expression. In a study on urothelial bladder cancer patients, co-occurrence of THOR hypermethylation and *TERTp* mutation was a marker of higher risk of disease recurrence and progression (74). Likewise, a study on melanoma patients showed a similar effect on reduced recurrence-free survival (75). These and other examples show that *TERTp* mutation coupled with THOR hypermethylation is a better marker of disease progression than *TERTp* mutation alone. Nevertheless, it should be noted that THOR hypermethylation does not associate with progression in a small group of cancers such as esophageal cancer, meningioma or pituitary adenoma (76).

Another interesting issue is the possible interplay between *TERTp* mutation, methylation, and histone modifications, which constitute yet another epigenetic mechanism affecting chromatin accessibility. A study by Stern et al. on monoallelic cancers showed that cancers without a specific *TERTp* mutation at −124 from the TSS had promoter hypermethylation, which was accompanied by repressive histone H3K27me3 methylation, leading to gene inactivation. They hypothesized that presence of this mutation coupled with low *TERTp* methylation discourages H3K27me3 histone methylation in transcriptionally active *TERT* (70). Interestingly, one study showed that *TERTp* hypermethylation was present in both melanoma and normal skin cells. However, only in melanoma cells with *TERTp* mutation did this hypermethylation correspond to increased *TERT* expression and chromatin accessibility (77). A further study by McKelvey et al. on thyroid cancer cell lines heterozygous for *TERTp* mutation demonstrated conclusively that *TERTp* methylation was allele-specific, whereby *TERTp* with mutation was significantly less methylated than wildtype promoter. Moreover, MYC, a transcription activator, bound only to the hypomethylated mutated *TERTp*, resulting in monoallelic expression (MAE) in heterozygous cells (29). MAE is one of two TERT expression categories as described by Huang et al., the other being biallelic expression (BAE, both alleles transcriptionally active). These two expression patterns appeared to be specific for many cancers, although some cancers exhibited variation between MAE and BAE in differed cell lines (34). However, a later study by Rowland et al. showed that this simple classification into MAE and BAE-specific cancer cell lines does not sufficiently describe the complex nature of *TERT* expression. In a study conducted on a single cell-level, they found great heterogeneity in TERT expression between various cells, within both the cell lines described as MAE, and those described as BAE by Huang et al. (78).

### micro-RNA

Most recent studies focus on *TERT* regulation at the transcriptional level. Meanwhile, post-transcriptional regulation by microRNAs (miRNAs), has not been expensively studied. miRNAs are a class of small non-coding RNAs (~22–24 nucleotides) (79). miRNA recognition sites are typically located in 3′ untranslated regions (3′UTRs) of mRNA (**Figures 1** and **2**). miRNAs binding to 3’UTR generally silences the transcript, thus reducing gene expression. miRNAs are ubiquitous elements of gene regulation, and control many different biological processes. In cancer, miRNAs function as gene regulatory molecules, acting as tumor suppressors or oncogenic drivers (18, 80).

Various miRNAs are known as regulators of *TERT*. In particular let-7g-3p, miR-128, miR-133a, miR-138-5p, miR-498, miR-541-3p, and miR-1182, downregulate expression of *TERT* and telomerase activation (18, 81). Functional analyses indicated that overexpression of miR-138-5p and miR-422a significantly inhibit *TERT* expression through interaction with *TERT* 3’UTR in colorectal cancer cells (79, 82). Moreover, miR-138-5p represses *TERT* protein expression in human anaplastic thyroid carcinoma and cervical cancer cells (79, 83). Likewise, miR-1182, miR-1266, miR-532, miR-1207-5p, and miR-3064 suppress gastric, bladder, ovarian cancer growth and invasion by binding to the *TERT* 3’UTR (10, 79, 84, 85). Furthermore, miR-128 was found to control TERT expression in HeLa and teratoma cell lines (81, 86).

miRNAs can also regulate *TERT* indirectly by controlling expression of various transcription factors. Accordingly, c-MYC, a major regulator of TERT, was regulated by miR-494 and miR-1294 in esophageal squamous cell carcinoma and pancreatic cancer. Additionally, c-MYC and FoxM1 were targeted by a known tumor suppressor, miR-34a, causing senescence in cells (18). Interestingly, the study of Lassmann et al. suggested that *TERT* is able to regulate miRNA levels at the early phase of miRNA processing. They demonstrated that deletion of *TERT* resulted in a decrease of most mature miRNAs (87).

## Transcription Factors

### Transcriptional Activators

*TERTp* contains binding sites for a huge number of transcriptional activators and repressors that directly or indirectly regulate gene expression. Multiple pathways, such as RAS/RAF/MEK/MAPK, PI3K/Akt/mTOR, IKK/NF-κB, transforming growth factor β/Smads, PKC, and the JAK-STAT pathway regulate *TERT* expression and telomerase enzymatic activity (88). In fact, most transcription factors have been identified as possible *TERT* gene regulators, such as protein kinases, growth factors, and oncogenic proteins. Canonical positive regulators of *TERT* transcription include the oncogene c-MYC, Sp1, NF-κB, STAT family of proteins, AP-2, and GSC. These activatory transcription factors will be described in detail in the following section.

*MYC* encodes a basic helix-loop-helix leucine zipper (bHLH-LZ) transcriptional factor called c-MYC (89, 90). The *MYC* gene family regulates expression of genes implicated in many processes, such as proliferation, cell growth, differentiation, self-renewal, apoptosis (91, 92). It is essential for embryonic development and it is expressed in normal somatic cells. There are several ways for healthy cells to control MYC levels, such as targeted degradation by the ubiquitin-proteasome system (92). Chromosome translocations, gene amplification, retroviral insertion or mutations of *MYC* gene are tumorigenic in mice and correlate with development of most human cancers (93, 94). c-MYC functions is dependent on heterodimerization with MAX (90, 95). While *MYC* gene contains a transcription activation domain, no such regulatory domain has been reported for MAX (96). The c-MYC/MAX heterodimers can bind to specific DNA sequences located within the core promoter region, known as E-box motifs (5′-CACGTG-3′), thus activating various genes (90, 92). c-MYC activates telomerase by inducing expression of *TERT* (90, 94). In addition, *TERT* is responsible for maintenance of c-MYC levels and regulates c-MYC proteasomal degradation (97).

The core promoter of *TERT* also contains specificity protein 1 (Sp1) binding sites that are necessary for *TERT* expression. Sp1 belongs to the family of nuclear proteins called Sp/KLF (specificity protein/Krüppel-like factor) that binds GC-(GGGGCGGGG) and GT-(GGTGTGGGG) rich elements (98, 99). It is one of the best characterized transcriptional activators of housekeeping genes and other TATA-less genes (89, 99). Sp1 regulates processes such as inflammation, carcinogenesis, senescence, hormonal activation, apoptosis and angiogenesis (98). Transcriptional activity of Sp1 is regulated by a few post-translational modifications (glycosylation, acetylation, phosphorylation) and by direct interaction with other proteins, including other transcription factors, nuclear factors, oncogenes, and tumor suppressors. Sp1-silencing completely inhibits telomerase activity by suppressing *TERT* expression, leading to apoptosis. Furthermore, mutations in Sp1 binding sites (GC‐boxes) significantly decrease transcriptional activity of *TERTp*, suggesting that Sp1 protein is involved in *TERT* transcription (100). Some reports indicated that cooperation between Sp1 and c-MYC drives cell type-specific *TERT* expression. This is further substantiated by the fact that normal cells have lower levels of Sp1 and c-MYC than cancer cells. However, Sp1 would be a weak candidate for a biomarker of cancer‐specific *TERT* expression because of its ubiquitous expression in normal cells (89, 100).

NF-κB is well known for playing a major role in inflammation, tumorigenesis, cytokine and chemokine expression, stress regulation, cell division and transformation (101, 102). NF-κB regulates expression of apoptosis inhibitors. The NF-κB signaling pathway is a master regulator of *TERT* activation in cancer cells. It initiates expression of *TERT* by binding to either of two potential motifs in *TERTp* (101). Additionally, *TERT* can directly regulate expression of NF-κB-dependent genes through binding to the p65 subunit. Studies have demonstrated that telomerase can directly regulate recruitment to promoters of NF-κB target genes, such as those encoding interleukin-6 (IL-6) and tumor necrosis factor alpha (TNF‐α) that are critical for inflammation and cancer progression (103).

The signal transducer and activator of transcription (STAT) family of cytoplasmic proteins are direct mediators of signaling from the extracellular environment to the nucleus (104). Seven STAT proteins have been identified as STAT 1–4, 5A, 5B, and 6 (105–107). They are normally inactive, but can be activated by phosphorylation. Of the seven human STAT encoding genes, STAT3 has drawn the most interest for its association with a wide variety of human cancers (104, 108).

In addition, these proteins are able to regulate *TERT* expression in tumor and normal cells (104). *TERTp* contains binding sites STAT3 and is overexpressed in prostate, breast, head, neck, and hematologic cancers, which implicates STAT3 as an important anticancer target (105).

The adipocyte protein 2 (AP-2) family of transcription factors contains five isoforms: AP-2α, AP-2β, AP-2γ, AP-2δ, and AP-2ϵ (109, 110). They are encoded by the *FABP4* gene. These isoforms have a major role in gene regulation and have different biological functions. They are required for morphogenesis during embryonic development (109). AP-2β specifically binds to the *TERTp* and activates telomerase in human cancer cells, but not normal cells. Two E-box sites in a 320-bp region of *TERTp* (320 bp upstream of the translational ATG site) have been observed to regulate promoter activity in human rhabdomyosarcoma cells (110).

A recent showed that goosecoid homebox protein (GSC) may be a new potential activator of *TERT* expression (49). It is normally involved in embryonic development and interacts with TGF-β and Wnt/β-catenin signaling pathways, which are implicated in tumor invasion (111). It was found to be overexpressed and to correlate with metastasis in patients with breast carcinoma (112), and was also associated with poor prognosis and chemoresistance in ovarian carcinoma (111). An analysis of *TERTp* areas with locally decreased methylation in thyroid cancer cells revealed a GSC biding site. GSC is a TERT activator and was variously expressed in both thyroid cancer and normal thyroid cells. Additionally, GSC was overexpressed in thyroid cancer (49).

### Transcriptional Repressors

Transcriptional repressors are proteins that attach to DNA at specific silencer sites and block transcription of nearby genes. In the following section, we are going to briefly discuss repressors that have been shown to downregulate *TERT* transcription, such as MAD1/2, p53, WT1, CTCF, and MZF-2.

The mitotic checkpoint is a crucial mechanism in maintaining chromosomal stability. It guarantees precise chromosome segregation by delaying separation of replicated sister chromatids (113, 114). Mitotic arrest deficient 1 (MAD1) is a major element of the mitotic checkpoint, and it recruits its binding partner MAD2 to nuclear pores (113, 115). During mitosis, MAD1 localizes to unattached kinetochores, where it serves as a docking site for MAD2. Kinetochore-bound MAD1–MAD2 act as a catalyst for conformational change of free MAD2 (114, 116). MAD1 upregulation serves as a marker of poor prognosis, as it tends to be overexpressed in cancers (116). Upregulation of MAD1 leads to chromosomal instability and resistance to microtubule poisons that are currently used as chemotherapeutic agents (116). MAD1 is recognized as an important cellular antagonist of c-MYC (117, 118). In addition, c-MYC and MAD1 are involved in regulation of *TERT* expression because they bind to the same promoter sites (E-box) to activate *TERT* expression (119). There two E-boxes in *TERTp*, and both of them constitute binding sites for c-MYC/MAX or MAD1/MAX heterodimers (117, 120). A switch from c-MYC/MAX to MAD1/MAX, triggers decrease in H3 and H4 histone acetylation at *TERTp* (119, 120).

p53 is the best known human tumor suppressor which is a member of a larger p53 family of tumor suppressors (121, 122). Other than p53, this family also includes p63 and p73 (123, 124). p53 acts primarily as an inducer of cell cycle arrest, cell differentiation, senescence, and apoptosis in response to numerous intrinsic and extrinsic stress signals (122, 125). It has a major role in the control of genomic stability, DNA replication, and DNA repair. The p53 encoding *TP53* gene is mutated in approximately 50% of human cancers. *TERTp* contains two p53 binding motifs (123). Several findings showed that p53 suppresses telomerase activity by inhibiting *TERT* expression (125). This inhibition may be caused indirectly, by an interaction between Sp1 and overexpressed p53 (125, 126). Furthermore, this inhibition of *TERT* could be possibly independent of other p53 functions, such as those associated with apoptosis (125).

Another protein implicated in inhibition of *TERT* is the Wilms’ tumor 1 (WT1) tumor suppressor (125). It contains four zinc fingers and an RNA-binding protein that directs the development of several organs (heart, diaphragm) and genitourinary tissues (127, 128). It is normally expressed in kidney, testes, ovaries, and spleen (129). Most neoplasms, including lung carcinomas, renal cell carcinoma, pediatric sarcomas, and breast, ovarian, colon, melanoma, and pancreas cancers, and exhibit a possible oncogenic activity of WT1 (130, 131). In addition, it is overexpressed in most acute myeloid leukemia patients, and is considered to be an independent marker of minimal residual disease (132). A WT1 binding site is located in *TERTp* (−352 upstream of the TSS), and its mutation significantly reduced telomerase activity and *TERT* mRNA expression in 293 embryonic kidney cells but not in HeLa cells (1, 89, 125). Additionally, WT1 inhibited *TERT* transcription during differentiation. This inactivation may influence activation of telomerase in the tumorigenesis phase. Furthermore, WT1 binding to *TERTp* suppresses c-MYC level at both protein, and mRNA level (1, 2).

CCCTC-binding factor (CTCF) is a zinc finger transcription factor which is ubiquitously expressed in human (133). Its binding sites are located in the first two exons of the *TERT* gene, and are located in a CpG island. Earlier studies showed that CTCF does not bind to TERT in telomerase-positive cells, which is correlated with methylation of exon 1 in these cells (134). Hypermethylation in this exonic region is common in most cancers, and CTCF is considered a major TERT repressor in normal cells. Methylation at specific CpG dinucleotides in exon 1 results in a change in secondary structure of DNA and creation of a four-strand structure known as G-quadruplex, which disrupts CTCF binding (135). Interestingly, CTCF was observed not to bind to TERT in normal thyroid tissue despite the presence of methylation, while thyroid cancer cell lines exhibited both partial methylation and CTCF binding (49).

The myeloid zinc finger protein (MZF)-2 is a Krüppel-like C_2_H_2_ zinc finger protein expressed predominantly in myeloid progenitor cells and involved in growth, differentiation, and tumorigenesis (136). The mechanisms involved in MZF-2-induced suppression of *TERTp* activity are still unclear (137). There are multiple binding sites for MZF-2 within the *TERTp* region, and upregulation of MZF-2 inhibits *TERTp* activity. This suggests a role for MZF-2 in transcriptional downregulation of *TERT* (125, 137).

## *TERT* Gene Polymorphisms

### Single Nucleotide Polymorphism

Single nucleotide polymorphisms (SNPs) have been described as being associated with increased risk for developing various cancers. They may be located both in intronic and exonic sequences of *TERT*, as well as in *TERTp*. Some common *TERT* SNPs found may modify survival and prognosis of certain cancers. A number of studies have recently been conducted to identify new SNP *loci* related to telomere length, which have shown a relationship between the risk of disease, its severity and the survival time in various cancers (138–140). In this section, we will discuss four common *TERT* polymorphisms that may be associated with gene expression.

Located at intron 2 of the *TERT* gene, rs2736100 A>C is a important non-coding SNP (141, 142). It has been associated with multiple cancers, especially with lung adenocarcinoma, which is characterized by significantly increased *TERT* gene expression, telomerase activity and gene copy number (143). Other solid cancers that are associated with this SNP include gliomas, bladder cancer, melanoma. rs2736100 has been identified as a major predisposing factor to sporadic and familial myeloproliferative neoplasms (MPNs), independently of the major diagnostic and molecular MPN subtypes. The *C* allele of rs2736100 and JAK2 46/1(GGCC) haplotype are major factors predisposing to MPN (141–143). Interestingly, the two alleles of rs2736100 seem to be associated with different types of diseases. While the *C* allele is primarily associated with cancers, the *A* allele, which is linked to shorter telomeres, is generally associated with predisposition to degenerative diseases (144). Furthermore, rs2736100 *C* is linked to increased blood cell count in the Japanese population (145).

Another *TERT* SNP, rs2853669 A>G is located in the *TERT*p region. It obstructs an ETS2 binding site, located close to an E-box. Previous studies showed that *TERTp* mutations creating a putative binding site for ETS, resulted in *TERT* upregulation and increased telomerase activity, while mutations at the ETS2 binding site suppressed c-MYC binding to the E-box (146, 147). Studies on rs2853669 showed that it is significantly associated with poor survival and increased cancer risk rate in hepatocellular carcinoma patients (146). In contrast, it was also observed to correlate with improved survival in patients with clear cell renal cell carcinoma, melanoma and glioblastoma (148). The *C* variant of this functional polymorphism results in decreased telomerase activity. Several studies suggest that rs2853669, in the presence of certain *TERTp* mutations, may also affect development of cancers (149). It was reported that it could influence telomere length and telomerase activity (150, 151). Furthermore, a study by Rachakonda et al. demonstrated that, in patients with urothelial bladder carcinoma, *TERT* rs2853669 may correlate with survival, prognosis, and tumor recurrence (152).

The two SNPs described above were located in non-coding regions of *TERT*, there are however also SNPs situated in exonic regions, of which rs2736098 G>A is a notable example. It is a synonymous A305A substitution located in exon 2, and was found to correlate with telomere length (153). Genotype *GG* was found to be associated with longer telomeres and decreased cancer susceptibility in patients with renal cell carcinoma (154). In another study, Xiao et al. showed that Chinese males harboring allele rs2736098 *A* had a greater risk of developing lung cancer than those with allele *G* (155). Allele *A* was also found to be significantly associated with risk of bladder cancer in the North Indian population (156). Further studies showed that it may impact risk for many other cancers, such as breast, esophageal, prostate, and basal cell carcinoma (153, 157).

### Variable Number of Tandem Repeats Polymorphism

It was demonstrated that *TERT* may be regulated *via* a variable number of tandem repeats (VNTR) polymorphism named MNS16A (**Figure 1**). It is located upstream of promoter region of an antisense *TERT* transcript. Depending on the number tandem repeats, promoter activity is affected differently. There are two MNS16A variant alleles: short (*S*) and long (*L*). The *L* allele correlates with higher promoter activity in the antisense strand and increased expression of the antisense *TERT* transcript. This increased expression of antisense *TERT* leads to silencing of functional *TERT* (158). As a result, the *S* allele is associated with higher telomerase activity, while *LL* homozygotes have lower telomerase activity (158). Our previous work showed that the *S* variant was more frequent in non-Hodgkin’s B-cell lymphoma patients how did not respond to treatment, as well as those with intermediate/high International Prognostic Index (159). In contrast, the *S* variant was less frequent in chronic lymphocytic leukemia patients with high disease stage (160).

## Alternative Splicing

TERT regulation is a multifarious process, which involves not only the transcriptional mechanisms described in the previous sections, but also posttranscriptional ones. This includes pre-mRNA alternative splicing of the *TERT* gene (161–163). There as many as 22 potential alternative splicing sites in the *TERT* gene, but the function of many of them is unclear (164–168). One of the most commonly studied splicing sites are deletions at two sites, α and β (**Figure 2**). The β splice site results in a major deletion (182 bp) and creates a non-functional, truncated protein. The α splice site generates a smaller (36 bp) deletion, which produces an impaired protein. Both of these splice sites result in TERT proteins that are incapable of telomere elongation (169–172). In many cancers, the full length TERT transcript (α+β+) correlated with tumor development and shorter survival in patients (173). However, the α variant alone is known to cause decreased telomerase activity and shorter telomeres, while the β splice variant was reported to not only inhibit telomerase activity but also the ability of cancer cells to induce apoptosis (174, 175). Another splice *TERT* variant may be generated by a deletion of exons 4–13, resulting in an inactive protein lacking its catalytic domain. This deletion was observed in both telomerase-negative and -positive cells, and was associated with increased cell proliferation (6).

## Involvement of TERT in Non-Telomere-Related Mechanisms

In the previous sections, we described *TERT* regulation and telomerase reactivation mechanisms that are involved in telomere maintenance. Telomere-related functions of TERT, also known as canonical, may likewise entail prevention of chromosome fusions (176, 177). However, telomerase also has non-canonical (telomere-independent) roles (**Figure 1**). These roles can be grouped into two broad categories: a) involving telomerase activity but not telomere elongation and b) involving neither telomere elongation nor telomerase activity (177). The telomere-independent roles contribute to the regulation of metabolic mechanisms, epigenetic regulation of chromatin, stress response, RNA silencing, signal transduction pathways (Wnt and c-MYC signaling pathways), enhanced mitochondrial function, cell adhesion, and migration (176, 178, 179).

TERT is found in cytoplasm and mitochondria, alongside its usual nuclear localization (176, 180) (**Figure 1**). In humans, mice and rats, TERT contains two specific targeting sequences that regulate its transport in and out of organelles: a nuclear targeting signal sequence, and a mitochondrial targeting sequence (181). In inactive CD4^+^ lymphocytes, TERT is mainly cytoplasmic but after activation it is transported to the nucleus in a process controlled by the kinase Akt (182). Additionally, shuttling TERT out of the nucleus may be promoted by oxidative stress, and this mechanism is dependent on phosphorylation of tyrosine 707 by Src kinase. Translocation of TERT into mitochondria improves mitochondrial potential which eventually leading to cancer cell survival (183). The extra-nuclear TERT functionalities are generally thought of as non-telomere related, i.e. non-canonical, and will be described below (179).

Cytoplasmic TERT exhibits many functions, including interacting with signaling pathways such as Wnt/β-catenin signaling. In addition, TERT binds to stress particles under non-stress conditions, and in lymphocytes, it is stored outside the nucleus without stimulation. TERT may also form a part of a TERT–NF-κB subunit p65 complex, which can move from the cytoplasm to the nucleus in multiple myeloma cells, upon TNF‐α induction (184). NF-κB, in turn, controls expression of a variety of genes involved in inflammation, immune responses, and cell differentiation (179). Zhou et al. demonstrated that the endoplasmic reticulum transiently activates the expression of *TERT* in cancer cell lines (185).

As much as 10–20% of total TERT is localized in mitochondria (176, 179). Therein, TERT binds to mitochondrial DNA (mtDNA) and improves respiratory chain activity, protecting mitochondrion from environmental damage and decreasing reactive oxygen species (mtROS) production (180, 186). mtROS production leads to mitochondrial damage and telomere shortening. Neutralization of mtROS does not recover the mitochondrial function but reduces telomere shortening (187). Additionally, telomere and mitochondrial disfunction is mediated by p53, which induces growth arrest, senescence and apoptosis in cells (188). TERT import depends on membrane potential and it is located close to the inner membrane (181). *TERT* binds to mtDNA in the region coding for NADH ubiquinone oxidoreductase subunits 1 (ND1) and 2 (ND2) and protects mtDNA from environmental damage (181). Mitochondrial TERT plays a role in decreasing apoptosis and improving mitochondrial membrane potential. Furthermore, it has unusual DNA- and RNA-dependent RNA polymerase activities, upon interaction with tRNAs (189). TERT can also interact with mitochondrial RNA processing endoribonuclease (RMRP) and use the RNA-dependent RNA polymerase to synthesize dsRNA. Mutations in RMRP can interfere with RMRP-TERT binding, contributing to pleiotropic syndrome cartilage–hair hypoplasia (190).

## TERT as a Potential Therapeutic Target

The unique feature of telomerase is its low or nonexistent expression in somatic cells, but overexpression in most cancer cells (191). Thus, telomerase and other telomere components offer a highly attractive diagnostic and prognostic biomarker of cancer and a target for development of therapeutics. Several strategies have been devised to target telomerase functions: telomerase inhibition, telomerase peptide vaccines, and suicide gene therapy. Epigenetic processes were suggested as another promising target for therapeutic purposes (192). Some of these are already used in treatment of patients as part of clinical trials (193).

*TERT* inhibition has been regarded as a promising therapeutic strategy, as earlier *in vitro* studies showed that TERT silencing cell proliferation (194, 195). An early approach was to design compounds that would interact with DNA at the 3’ overhang, stabilizing telomeric G-quadruplex secondary structures, and thus blocking telomerase access to DNA. Telomestatin, BRACO-19, RHPS4, TMPyP4 are some of the most commonly studied G-quadruplex binding proteins (191, 196, 197). Telomestatin (OBP-301) is a natural product isolated from *Streptomyces anulatus* (198). The primary mechanism of telomestatin action involves a highly specific interaction with the G-quadruplex to stabilize its structure (199). These DNA-binding compounds are now less popular due to discovery of better molecular strategies, such as targeting the TERT active site directly. Studies on such inhibitors led to discovery of 2-[[(E)-3-naphthalen-2-ylbut-2-enoyl]amino]benzoic acid (BIBR1532), which inhibits telomerase by binding non-competitively to the TERT active site (197, 200). This binding leads to increased oxidative stress and decreased nitrogen monoxide bioavailability in favor of H_2_O_2_. However, BIBR1532 has not yet progressed to clinical tests (201). Aside from synthetic compound, various naturally occurring compounds, such as allicin (from garlic), curcumin (from turmeric), silibinin (from thistle), and epigallocathechin gallate (EGCG, from tea) were found to have telomerase inhibitory properties (202). A synthetic, more stable derivative of EGCG, MST-312, was shown to inhibit telomerase in various cancer, although its mechanism of action remains unknown (203–205).

Some peptide vaccines can possibly target the telomerase active site (199). GV1001 (KAEL-GemVax Co. Ltd., Gangnam-gu Seoul, Republic of Korea) is the only such vaccine to enter clinical trials (206). Its structure is based on a peptide sequence from TERT active site and it capable of binding multiple HLA class II molecules. It functions by stimulating tumor-reactive CD8^+^ and CD4^+^ T-cell immunity specific for TERT (199, 207, 208). GV1001 is used in treatment of patients with advanced stage melanoma, lung, hepatocellular carcinoma and pancreatic cancer (196). Two other TERT-based peptide vaccines, p540 and p675 were also observed to elicit TERT-specific cytotoxic T cell HLA-A*02:01- restricted immunity (208, 209). Other TERT-based vaccines are composed of more than one separate peptide sequence. An example of such a vaccine is GX301, composed of four peptides. This multi-peptide character means that it recognizes more HLA haplotypes, binding to both class I and II HLA molecules (210). GX301 is currently (October 2020) in phase II of a clinical trial on patients with prostate cancer (211). GRNVAC1 is a dendritic cell vaccine, which was created by transfecting dendritic cells with mRNA encoding TERT-chimeric protein, and then returning the transfected cells to the patient (196). These cells would then target telomerase-expressing tumor cells. The clinical trial is in phase I/II, and the vaccine is currently used in treatment of patients with metastatic prostate cancer (196, 207).

Another strategy are the suicide gene therapies. They include oncolytic virotherapy, the predominantly used strategy to treat cancer, which has potential to specifically lyse the tumor, and not healthy cells. This approach involves adenoviruses replicating selectively in cancer cells, and subsequently killing them (212). This viral system relies on the highly active *TERC*/*TERT* promoter controlling expression of a bacterial protein nitroreductase. Neither this nor any other suicide gene therapy has entered into clinical trials (193).

Recent studies increasingly suggest that epigenetic mechanisms may be targeted in new therapeutic strategies. Chidamide, an inhibitor of the enzyme histone deacetylase, was shown to decrease telomerase expression through miR-129-3p up-regulation in non-small cell lung cancer cells. This leads to subsequent ROS accumulation and subsequent cell cycle arrest (213). Epigenetic mechanisms may also be exploited in potential therapies using personalized approach. A study on effects of all-trans retinoic acid (ATRA) in treatment of ovarian carcinoma patients showed that the efficacy of therapy correlated inversely with methylation level of *TERTp*. This was of particular interest in a large subgroup of serous ovarian carcinoma patients, who had hypomethylated *TERTp*, and could therefore be treated effectively with ATRA (214).

As shown by the examples described above, telomerase is an attractive target for cancer immunotherapy. The main advantage of TERT is its high cancer-specific expression. Results from clinical trials have been encouraging, because of the safety and good tolerability of telomerase inhibitors (215). As a final point, it should be noted that using just one type immunotherapy may not suffice to eliminate cancer cells. Therefore, new studies should focus on strategies integrating various types of therapies (216).

## Summary

*TERT* is normally actively transcribed only in early embryonic development and in cells with high proliferative potential, while it is inactive in most somatic cells in adults. However, in most cancers, *TERT* undergoes reactivation, and by extending telomeres (the canonical function of TERT) it contributes to cancer formation and progression. There are many regulatory mechanisms involved in telomerase reactivation and adjustment of *TERT* expression, among which *TERTp* mutation is perhaps the most important. Other major TERT regulation mechanisms (also known as telomere maintenance mechanisms) are: chromosome rearrangements, methylation, miRNA interference, binding of transcription factors, genetic polymorphism, and alternative splicing. Some of these mechanisms may interact with each other, having a synergistic effect on TERT expression. Aside from the better-known telomere lengthening function, TERT also has many secondary, telomere-independent roles (non-canonical functions of TERT). Taking in to account its major importance in cancer, TERT has become a target of various therapeutic strategies in cancer treatment and continues to be an interesting object of research.

The following features of TERT described in this manuscript can be highlighted:

- TERT is a functional catalytic protein subunit of telomerase, which lengthens telomeres by adding short DNA repeats, consequently averting chromosomal instability;
- Its regulation is a multifarious process where both transcriptional and posttranscriptional mechanisms are involved;
- TERT is also a major component of various oncogenic signaling pathways, and its overexpression often contributes to tumorigenesis;
- *TERT* gene is often overexpressed in cancers, and this overexpression can be induced by a variety of mechanisms, such as: *TERT* gene amplification, *TERT* gene polymorphism, *TERTp* mutation and methylation, and miRNA interference, alternative splicing of the *TERT*;
- Aside from its primary nuclear localization, TERT can also be transported to cytoplasm and mitochondria;
- It has many non-canonical, i.e. telomere-unrelated, functions these include: interaction with signaling pathways, stress protection, regulation of chromatin structure, binding to and protection of mitochondrial DNA;
- TERT and its gene may also act as an attractive target for therapeutic interventions with a diagnostic and prognostic impact.

## Author Contributions

MD and KB-K contributed to the conception and design of the review, drafted, and finalized the manuscript. BW contributed to the conception and design of the review and its draft version. PŁ contributed to the writing, reviewing, and editing of the final version of the manuscript. TK contributed to the final version of the manuscript and drew all the figures. All authors contributed to the article and approved the submitted version.

## Funding

This work was supported by the TARGETTELO project No. STRATEGMED3/306853 from the National Centre for Research and Development, Warsaw, Poland.

## Conflict of Interest

The authors declare that the research was conducted in the absence of any commercial or financial relationships that could be construed as a potential conflict of interest.
